# Potential role of epicardial adipose tissue as a biomarker of anthracycline cardiotoxicity

**DOI:** 10.1186/s13244-021-01069-4

**Published:** 2021-11-06

**Authors:** Caterina Beatrice Monti, Simone Schiaffino, Maria Del Mar Galimberti Ortiz, Davide Capra, Moreno Zanardo, Elena De Benedictis, Alberto Gianluigi Luporini, Pietro Spagnolo, Francesco Secchi, Francesco Sardanelli

**Affiliations:** 1 Department of Biomedical Sciences for Health, Università Degli Studi Di Milano, Via Mangiagalli 31, 20133 Milano, Italy; 2 Unit of Radiology, IRCCS Policlinico San Donato, Via Morandi 30, 20097 San Donato Milanese, Italy; 3 Unit of Medical Oncology, Policlinico Di Monza, Via Amati 111, 20900 Monza, Italy; 4 Unit of Medical Oncology, IRCCS Policlinico San Donato, Via Morandi 30, 20097 San Donato Milanese, Italy

**Keywords:** Anthracyclines, Cardiotoxicity, Breast neoplasms, Biomarkers, Tomography, X-ray computed

## Abstract

**Background:**

We investigated the radiodensity of epicardial (EAT), subcutaneous (SAT), and visceral adipose tissue (VAT) before and after treatment with anthracyclines in a population of breast cancer (BC) patients, and in controls not treated with anthracyclines, to detect a potential role of EAT density as a biomarker of changes related to chemotherapy cardiotoxicity.

**Methods:**

We reviewed BC patients treated with anthracyclines who underwent CT before (CT-t_0_) and after (CT-t_1_) chemotherapy, and age- and sex-matched controls who underwent two CT examinations at comparable intervals. On non-contrast scans, EAT was segmented contouring the pericardium and thresholding between -190 and -30 Hounsfield units (HU), and SAT and VAT were segmented with two 15-mm diameter regions of interest thresholded between -195 and -45 HU.

**Results:**

Thirty-two female patients and 32 controls were included. There were no differences in age (*p* = 0.439) and follow-up duration (*p* = 0.162) between patients and controls. Between CT-t_0_ and CT-t_1_, EAT density decreased in BC patients (-66 HU, interquartile range [IQR] -71 to -63 HU, to -71 HU, IQR -75 to -66 HU,* p* = 0.003), while it did not vary in controls (*p* = 0.955). SAT density increased from CT-t_0_ to CT-t_1_ in BC patients (-107 HU, IQR -111 to -105 HU, to -105 HU, IQR -110 to -100 HU,* p* = 0.014), whereas it did not change in controls (*p* = 0.477). VAT density did not vary in either BC patients (*p* = 0.911) or controls (*p* = 0.627).

**Conclusions:**

EAT density appears to be influenced by anthracycline treatment for BC, well known for its cardiotoxicity, shifting towards lower values indicative of a less active metabolism.

## Key points

- We retrospectively assessed epicardial adipose tissue (EAT) CT density in breast cancer patients.
- EAT CT density decreased after anthracyclines treatment.
- Lower EAT density after chemotherapy may be an indicator of lower cardiac metabolism.

## Introduction

Breast cancer (BC) is the most common malignancy in women, with estimates indicating that women in the European Union have a 1 in 11 chance of developing BC before 74 years of age [1]. While 5-year survival rates have risen to about 90% due to improvements in treatment and screening effectiveness, the incidence of BC is rising yet again [2]. Thus, a growing body of BC survivors is facing new health complications related to BC and its treatment, the most worrisome being cardiovascular disease, a leading cause of mortality in this population [3]. Indeed, many BC treatment options yield significant cardiotoxicity: anthracyclines and radiation therapy have long been linked to a dose-related development of myocardial fibrosis, whereas trastuzumab and immune checkpoint inhibitors have been associated to stochastic cardiotoxic effects [4]. Nonetheless, anthracyclines still represent a cornerstone of BC treatment, especially in patients with advanced disease or triple negative neoplasms [5]. In addition to the well-known toxic effects on cardiomyocytes, anthracyclines are metabolised by adipose tissue and may produce shifts in adipocytes activity in different adipose tissues [6].

Epicardial adipose tissue (EAT) is a layer of beige adipose tissue located between the epicardium and visceral pericardium, which enacts both mechanical and biochemical functions to support the myocardium [7]. In particular, EAT provides a source of nutrition to the heart through the regulation of the local metabolism of free fatty acids and adipokines [8]. Therefore, dysregulations of EAT functions, macroscopically marked by an increase in EAT volume, have been related to an increased cardiovascular risk [9]. More recently, EAT density, measured via computed tomography (CT) in Hounsfield units (HU), has been proposed as a cardiovascular risk biomarker, displaying correlations with the onset of coronary artery disease and atrial fibrillation, as well as cardiac involvement in severe novel coronavirus diseases [10–12].

Given the crucial role of EAT in the development of cardiovascular disease, and the effects of anthracycline treatment on both adipose tissue and myocardium, we might hypothesise that variations in EAT density could be related to the cardiotoxic effects of anthracyclines. Thus, the purpose of our study was to assess the CT density of EAT before and after treatment with anthracyclines in a population of BC patients, and in controls who underwent CT examinations at comparable time intervals, to detect a potential role of EAT in changes related to chemotherapy cardiotoxicity. Pre- and post-treatment densities of subcutaneous adipose tissue (SAT) and visceral adipose tissue (VAT) were also assessed in the two populations, considering the potential confounding effect of treatment on systemic adipose tissue.

## Materials and methods

### Ethics committee

The local Ethics Committee (Ethics Committee of IRCCS Ospedale San Raffaele) approved this retrospective study (protocol code “CardioRetro”, number 122/int/2017; approved on September 14, 2017, and amended on February 10, 2021), and informed consent was waived due to the retrospective nature of the study.

### Study population

We retrospectively reviewed BC patients treated with an anthracycline-based chemotherapy regimen, and who underwent at least two chest CT examinations for disease staging or re-staging at our institution between March 2012 and June 2020, one acquired before the start of chemotherapy (CT-t_0_), and the other following the end of treatment (CT-t_1_). As CT-t_1_, we considered the examination at the longest available follow-up, at least 3 months after the end of chemotherapy, to avoid potential confounding from myocardial oedema due to chemotherapy myocardial toxicity. Each CT examination had to include one unenhanced scan. Patients who underwent radiation therapy to the left breast between CT-t_0_ and CT-t_1_ were excluded, because of potential confounders [13]. Moreover, we excluded all CT scans which presented artefacts that would hinder the segmentation of EAT, SAT, or VAT. Our patient population was already included in a study appraising the role of myocardial extracellular volume in anthracycline cardiotoxicity [14]. For each patient, we retrieved demographical, BC, staging, and treatment data.

As a control population, we selected age-matched women who had undergone chest CT examinations including at least one unenhanced scan for any indication except those pathologies yielding known or potential myocardial injury (such as pulmonary embolism or mediastinal lymphomas), at time intervals comparable to those in-between CT-t_0_ and CT-t_1_ in our population of patients, and who did not undergo chemotherapy with anthracyclines, radiation therapy, or any other cardiotoxic treatment.

### Image acquisition

Patients were studied using a 64-row CT scan (Somatom Definition, Siemens Medical Solution, Erlangen, Germany) with 120 kVp, tube current ranging from 100 to 200 mAs depending on automatic exposure control system (CARE Dose 4D, Siemens Medical Solution, Erlangen, Germany), rotation speed 0.5 s, pitch 1, B30f medium smooth for kernel reconstruction technique and abdominal CT window, or a 16-row CT scan (Emotion 16, Siemens Medical Solution, Erlangen, Germany) with 130 kVp, tube current ranging from 100 to 200 mAs depending on automatic exposure control system (CARE Dose 4D, Siemens Medical Solution, Erlangen, Germany), rotation speed 0.5 s, pitch 1, B30f medium smooth for kernel reconstruction technique. All patients underwent CT protocols based on their clinical status and indications, and all CT protocols included an unenhanced scan which was acquired with the above-mentioned parameters and was then used for adipose tissue segmentation.

### Image analysis

**Fig. 1:**
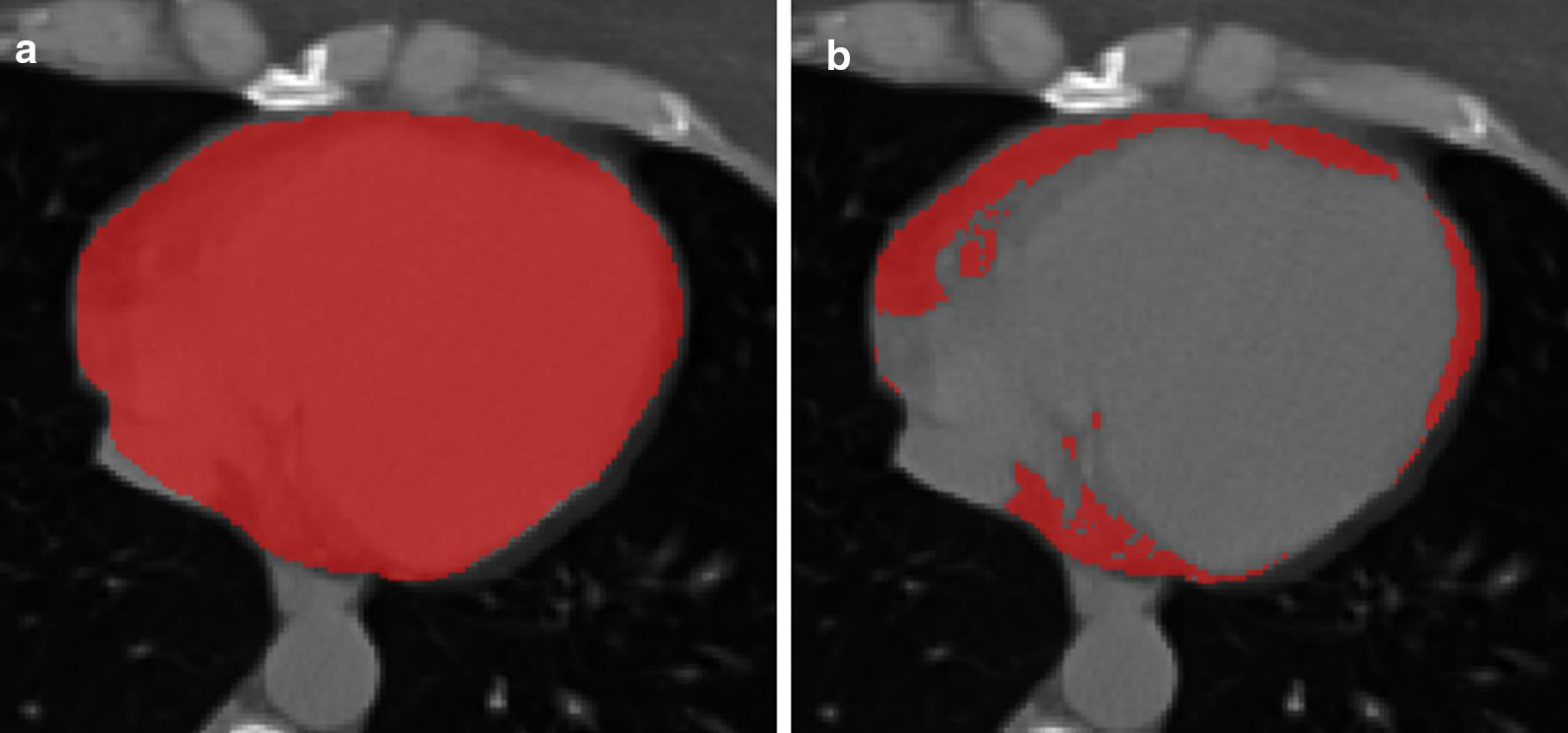
Epicardial adipose tissue segmentation. **a** contouring of the pericardium; **b** subsequent thresholding between -190 and -30 Hounsfield units

One reader, a medical student with 2 years of experience in cardiovascular CT performed image segmentation with the supervision of a more experienced reader (C.B.M.) with 5 years of experience in cardiovascular CT. Image segmentation was performed using ITK-SNAP Version 3.8.0 (www.itksnap.org [15]). For EAT assessment, the whole pericardium was contoured and the region of interest (ROI) generated from this segmentation was then thresholded between -190 and -30 HU, as already described [16]. An example of EAT segmentation is presented in Fig. 1. Afterwards, to appraise the reproducibility of EAT density, a third reader (MDMGO) with 3 years of experience in cardiovascular CT performed all EAT segmentations, following the same procedure.

**Fig. 2:**
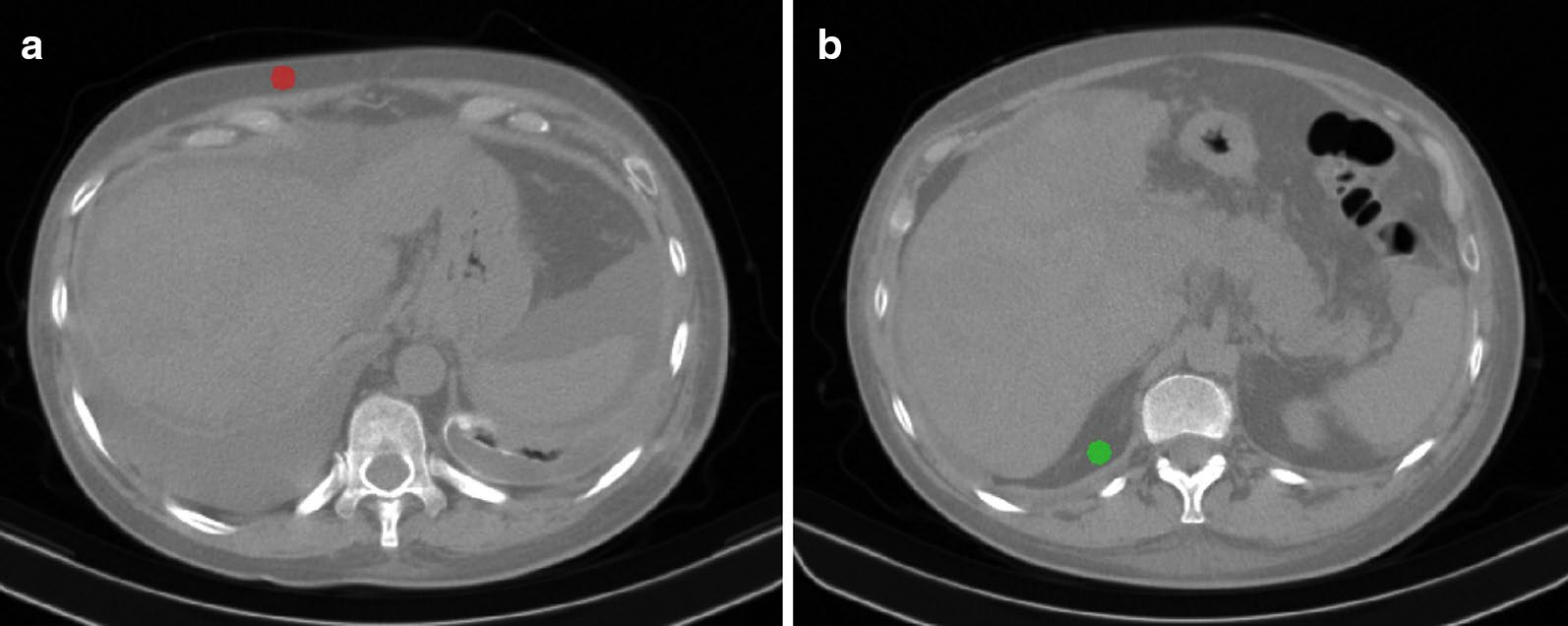
Segmentation of: **a** subcutaneous adipose tissue and (**b**) visceral adipose tissue by placement of round regions of interest with a 15 mm diameter

For the segmentation of SAT and VAT, the first reader placed two ROIs with a diameter of 15 mm in the context of abdominal SAT and VAT (Fig. 2). Such ROIs were then thresholded between -45 and -195 HU [17].

### Statistical analysis

Data distributions were assessed both visually and with the aid of the Shapiro–Wilk test. Normally distributed variables were reported as mean and standard deviation, whereas non-normally distributed variables were reported as median and interquartile range (IQR). Correlations were assessed with Pearson *r* or Spearman *ρ* according to data distribution normality, and resulting correlation coefficients were reported as proposed by Evans [18]. Differences between variables were assessed with t tests or Wilcoxon and Mann-Whitney *U*, according to data distribution normality. Reproducibility was appraised with Bland–Altman analysis and reported as bias and coefficient of repeatability. Statistical analyses were performed using Python 3.7.6, and *p* values below 0.05 were considered as statistical significant [19].

## Results

### Study population

Overall, 33 patients referring to our institution for BC treatment met the inclusion criteria for this study. However, one patient who received radiation therapy to the left breast was excluded, leading to a final study population of 32 patients having undergone an anthracycline-based chemotherapy regimen. All patients were females, and median age at CT-t_0_ was 55 years (IQR 48−68 years). All patients had a histologic demonstration of BC: 29/32 patients (91%) had infiltrating ductal carcinoma, 2/32 (6%) had a poorly differentiated carcinoma, and 1/32 (3%) had a neuroendocrine carcinoma. All patients had stage II or higher BC (10/32 (31%) stage II, 13/32 (41%) stage III, and 9/32 (28%) stage IV) and had undergone anthracycline-based chemotherapy with dosages adjusted to body surface area according to clinical guidelines: 26 patients received epirubicin with a total dose of 360 mg/m^2^, 4 patients received mitoxantrone with a total dose of 40 mg/m^2^, and 2 patients received 240 mg/m^2^ of adriamycin. Median left ventricular ejection fraction before chemotherapy was 66% (IQR 60−68%), and median time between CT-t_0_ and CT-t_1_ was 31 months (IQR 20−53 months). No patient treated with anthracyclines showed overt cardiotoxicity, with left ventricular ejection fraction remaining within normal range and displaying no significant decreases.

The 32 matched controls were referred to CT for colon cancer (16/32, 50%), liver disease (11/32, 34%), or evaluation of pulmonary lesions (5/32, 16%). Controls had a median age comparable to that of BC patients (55 years, IQR 48−67 years, *p* = 0.439) and had undergone the two CT examinations in comparable time frames (27 months, IQR 13−43 months, *p* = 0.162). Demographic data of patients and controls are reported in Table 1.

**Table 1:** Demographical and adipose tissue data for breast cancer (BC) patients undergoing treatment with anthracyclines and controls

	Breast cancer patients		Controls		*p*
N	32		32		-
Females (*n*, %)	32 (100)		32 (100)		-
Age (years)	55 (48 to 68)		55 (48 to 67)		0.439
Pathology (*n*, %)	Cancer stage		CT indication		
	II	10 (31)	Colon cancer	16 (50)	-
	III	13 (41)	Liver disease	11 (34)	-
	IV	9 (28)	Pulmonary lesions	5 (16)	-
CT-t_0_ EAT density (HU)	-66 (-71 to -63)		-64 (-74 to -60)		0.439
CT-t_0_ SAT density (HU)	-107 (-111 to -105)		-104 (-109 to -101)		0.030
CT-t_0_ VAT density (HU)	-107 (-112 to -102)		-106 (-113 to -97)		0.501
Interval CT-t_0_–CT-t_1_ (months)	31 (20 to 53)		27 (13 to 43)		0.162

CT computed tomography, CT-t_0_ baseline CT, CT-t_1_ follow-up CT, EAT epicardial adipose tissue, SAT subcutaneous adipose tissue, VAT visceral adipose tissue, HU Hounsfield units. Quantitative data are reported as median and interquartile range

Concerning the kVp of CT acquisitions in the BC cohort, 27/32 patients (84%) were acquired at 130 kVp and 5/32 (16%) at 120 kVp at CT-t_0_, without significant differences from CT-t_1_ when 28/32 patients (88%) were acquired at 130 kVp and 4/32 (12%) at 120 kVp (*p* = 0.999). Among controls, at CT-t_0_ 16/32 patients (50%) were acquired at 130 kVp and 16/32 (50%) at 120 kVp, whereas at CT-t_1_ 2/32 patients (7%) were acquired at 130 kVp and 30/32 (93%) at 120 kVp (*p* = 0.002).

### Epicardial adipose tissue

**Fig. 3:**
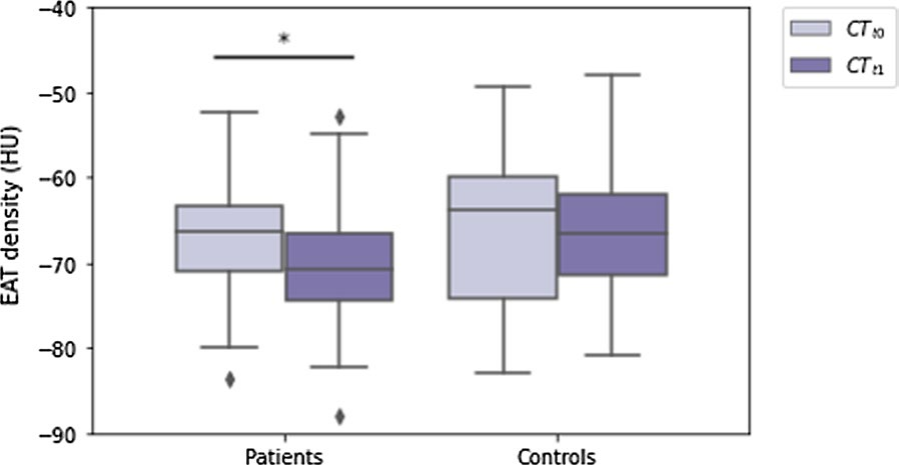
Boxplots depicting the distributions of epicardial adipose tissue density at the first (CT-t_0_) and second (CT-t_1_) computed tomography examination in patients with breast cancer who underwent treatment with anthracyclines, and in controls with no breast cancer who did not undergo cardiotoxic treatment. * denotes statistical significance

At CT-t_0_, patients receiving anthracyclines showed a median EAT density similar to that of controls (-66 HU, IQR -71 to -63 HU *versus* -64 HU, IQR -74 to -60 HU, *p* = 0.439). Between CT-t_0_ and CT-t_1_, EAT density decreased in these patients reaching a median value of -71 HU (IQR -75 to -66 HU, *p* = 0.003), while it did not vary significantly in controls, with a median CT-t_1_ density of -67 HU (IQR -71 to -62 HU, *p* = 0.955). The median difference in EAT density between CT-t_0_ and CT-t_1_ in BC patients receiving anthracyclines was -2 HU (IQR -5 to 0 HU). Such difference in EAT density did not display significant correlations with anthracycline dose (ρ = 0.004, *p* = 0.982), BC histology (ρ = 0.042, *p* = 0.818), pre-treatment ejection fraction (ρ = 0.144, *p* = 0.513), or patients’ age (ρ = 0.180, *p* = 0.324). EAT density distributions at CT-t_0_ and CT-t_1_ in patients receiving anthracyclines and controls are displayed in Fig. 3.

**Fig. 4:**
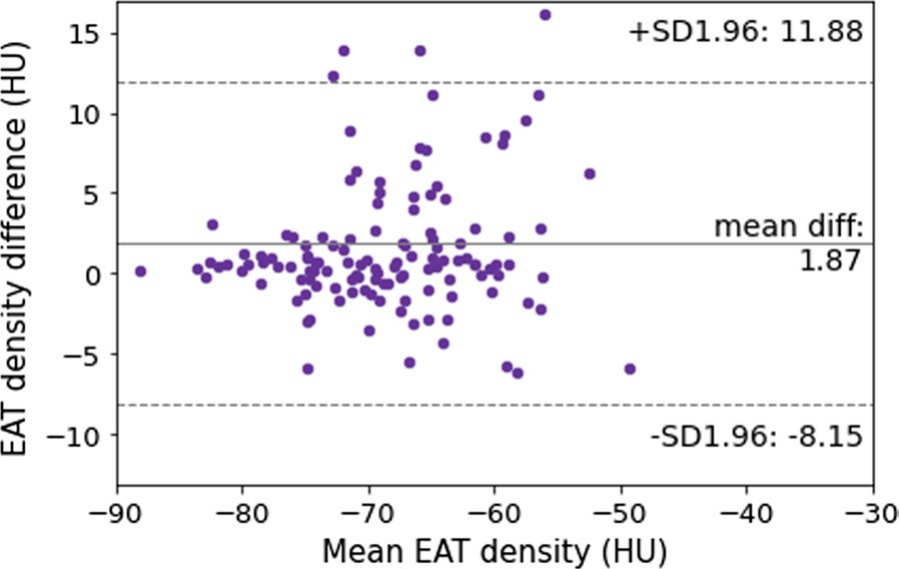
Bland–Altman plot for inter-reader reproducibility of epicardial adipose tissue (EAT) density

At Bland–Altman analysis, EAT density showed a bias of 2 HU, with a coefficient of repeatability of 10 HU. The Bland–Altman plot for EAT density reproducibility is displayed in Fig. 4.

### Subcutaneous and visceral adipose tissue

**Fig. 5:**
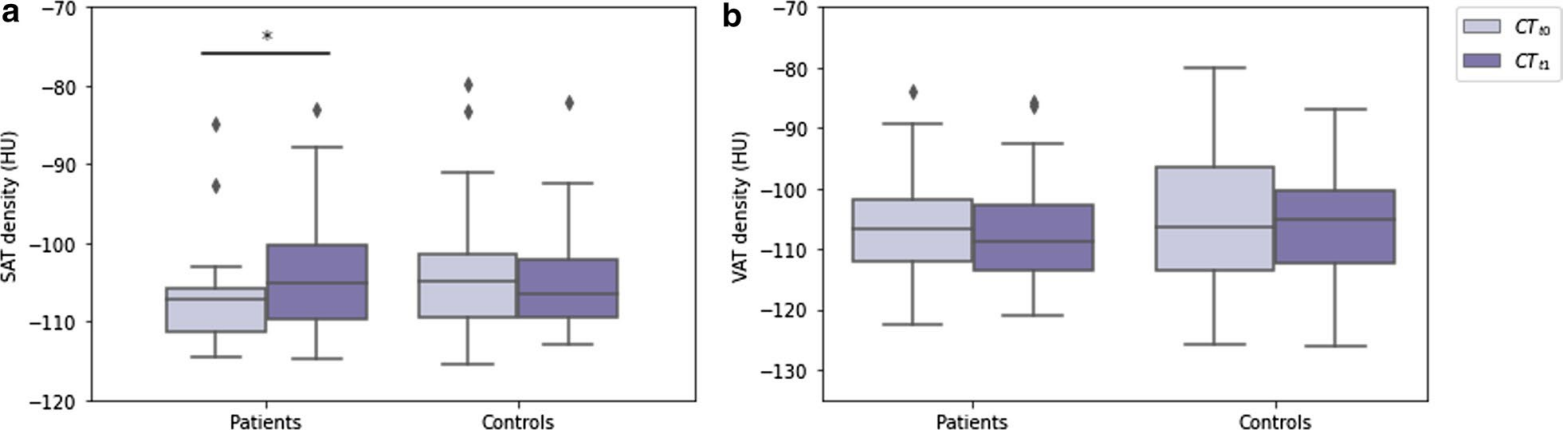
Boxplots depicting: **a** the distributions of subcutaneous adipose tissue and (**b**) visceral adipose tissue density at the first (CT-t_0_) and second (CT-t_1_) computed tomography examination in patients with breast cancer who underwent treatment with anthracyclines, and in controls with no breast cancer who did not undergo cardiotoxic treatment. * denotes statistical significance

At CT-t_0_, SAT density was lower in patients receiving anthracyclines than in controls (-107 HU, IQR -111 to -105 HU *versus* -104 HU, IQR -109 to -101 HU, *p* = 0.030). SAT density increased from CT-t_0_ to CT-t_1_ in these patients, reaching a median of -105 HU (IQR -110 to -100 HU, *p* = 0.014), whereas it did not change significantly in controls, with a median at CT-t_1_ of -106 HU (IQR -109 to -102 HU, *p* = 0.477). SAT density distributions at CT-t_0_ and CT-t_1_ in BC patients receiving anthracyclines and controls are displayed in Fig. 5.

At CT-t_0_, VAT density did not display significant differences between patients receiving anthracyclines and controls (-107 HU, IQR -112 to -102 HU *versus* -106 HU, IQR -113 to -97 HU, *p* = 0.501), and it did not change in either group with CT-t_1_ density values of -109 HU (IQR -114 to -103 HU, *p* = 0.911) in patients receiving anthracyclines and -105 HU, (IQR -112 to -100 HU, *p* = 0.627) in controls. VAT density distributions at CT-t_0_ and CT-t_1_ in patients receiving anthracyclines and controls are displayed in Fig. 5.

## Discussion

Given the potential cardiotoxicity related to treatment with anthracycline-based regimens, in this work we assessed pre- and post-treatment CT EAT density in BC patients, observing a significant decrease in EAT density which was not evident in controls, who were not treated with anthracyclines.

The median values of EAT density on unenhanced scans at CT-t_0_ for patients (-66 HU) and controls (-64 HU) observed in our study are slightly higher than those reported by previous studies, as that by Goeller et al. [11] who described a mean EAT density of -76.2 HU in asymptomatic patients undergoing cardiac CT for the assessment of coronary artery disease. This difference could be due to the different demographics: the average age of our patients (55 years) was lower than that of patients in the referenced work (60 years). Indeed, a study by Nerlekar et al. [16] outlined how EAT density decreases with age, with a mean decrease of 10 HU over about 4 years. Concerning SAT and VAT density, the median values observed in our patients and controls (-107 HU and -104 HU, respectively, for SAT, and -107 HU and -106 HU, respectively, for VAT) are relatively close to those reported by previous works [20, 21]. The lower SAT density at CT-t_0_ observed in our patients group compared to controls might be an indicator of the fact that BC patients may present with a less active, more lipidic adipose tissue, which could be linked to a higher risk of both BC cardiometabolic disease [22].

The decrease in EAT density observed in patients after chemotherapy with anthracyclines can be explained by two main hypotheses. First, as EAT plays a pivotal role in sustaining cardiac metabolism, myocyte necrosis and consequent cardiac fibrosis associated with anthracycline treatment could lead to a decrease in the metabolic activity of the myocardium, which could in turn lead to a decrease in EAT metabolism and consequential adipose tissue whitening [23]. Alternatively, the decrease in EAT density could be due to the lower metabolic requirements of the myocardium of cancer patients undergoing chemotherapy, who most often become less active due to disease- and treatment-related side effects [24]. Nevertheless, our control group was composed by patients with colon cancer, who undergo chemotherapy as well, albeit with considerably less cardiotoxic regimens, and who might too therefore prove less active [25]. Conversely, the concomitant increase in SAT density detected in patients undergoing anthracycline-based chemotherapy regimens might be due to an overall loss of adipose tissue observed in patients with advanced cancer, as all our patients presented with stage II or higher BC, which would actually lead to a loss of adipocytes lipids with a consequent increase in CT radiodensity [26]. Moreover, anthracyclines may be directly metabolised by subcutaneous adipocytes, which might sequester chemotherapeutic agents and thus lead to lower treatment efficacy in obese subjects, and the accumulation of anthracyclines metabolites along with the metabolic changes they provoke might produce a higher CT density [27]. The difference in kVp used in controls between CT-t_0_ and CT-t_1_ with lower values at CT-t_1_ further enhances the lack of decrease in EAT density, as lower kVp acquisition values have been correlated with lower adipose tissue density at CT [28].

The lack of correlations between the decrease in EAT density and anthracycline dosage might be explained by the fact that while anthracycline-related cardiotoxicity is indeed dose-related, not all patients treated with anthracyclines respond by developing cardiotoxicity with consequent myocardial necrosis and fibrosis, thus leading to a high interpersonal variability [29].

While our population was already involved in a previous work assessing the role of myocardial extracellular volume as a biomarker of anthracycline cardiotoxicity [14], such analyses require the presence of both unenhanced and contrast-enhanced CT scans including the heart and might therefore prove less widely feasible than the sole assessment of EAT density on unenhanced scans, hence providing an edge to the use of EAT density in this context.

Our study presents some limitations. First, all CT scans included in this study were non-electrocardiographically gated, and therefore, the movement of the heart might have led to motion artefacts and inaccurate voxel density values for EAT. Nevertheless, all segmentations of the pericardium were thresholded between -190 and -30 HU, thus only included voxels containing adipose tissue, and the median EAT density values in our patients and controls were only slightly higher than those reported by previous works, in line with trends reported in the literature [11]. Additionally, inter-reader reproducibility for EAT density proved high, with a small bias (2 HU) compared to EAT density magnitude. Second, we did not assess SAT and VAT density over one whole slice, but rather from single, round ROIs. Nevertheless, such ROIs were carefully placed in regions containing SAT and VAT, respectively, and subsequently thresholded, excluding all but adipose tissue according to voxel density. Third, our patient population is small and only includes patients with BC, which is not the only malignancy treated with anthracyclines [30]. However, the main aim of our work was an initial appraise potential effects on EAT, SAT, and VAT of anthracycline treatment, and further studies are warranted to better clarify the modifications in adipose tissue density caused by anthracyclines, and their relative associations with cardiotoxicity or other side effects. Lastly, due to the retrospective nature of this work we could not retrieve data concerning the metabolic status of patients, such as blood test results or morphometric information, at uniform times and intervals, and thus, we could not analyse the relationship between EAT density and such variables. Thus, further prospective studies are warranted to clarify these issues.

In conclusion, our results showed that variations in EAT CT density may reflect local changes secondary to anthracycline treatment. Thus, considering that unenhanced, non-gated CT scans are often part of the clinical workflow of BC patients who receive an anthracycline-based chemotherapy regimen, the evaluation of EAT density could be implemented in such setting to review its potential role as a biomarker of cardiotoxicity.

## Data Availability

The dataset used during the current study is available from the corresponding author on reasonable request.

## Abbreviations

BC: Breast cancer
CT: Computed tomography
CT-t_0_: Pre-chemotherapy CT scan
CT-t_1_: Post-chemotherapy CT scan
EAT: Epicardial adipose tissue
HU: Hounsfield units
IQR: Interquartile range
ROI: Region of interest
SAT: Subcutaneous adipose tissue
VAT: Visceral adipose tissue
